# Influence of meteorological factors on scrub typhus in Southeast China: a study across 100 districts in Jiangxi Province

**DOI:** 10.1186/s41182-025-00835-0

**Published:** 2025-11-07

**Authors:** Yanwu Nie, Yisheng Zhou, Shu Yang, Xiaobo Liu, Yibing Fan, Qinhan Jiang, Yong Liu, Yangqing Liu, Daiwei Zhang, Yuanan Lu, Hui Li, Lei Wu

**Affiliations:** 1 Jiangxi Provincial Health Commission Key Laboratory of Pathogenic Diagnosis and Genomics of Emerging Infectious Diseases, Nanchang Center for Disease Control and Prevention, Nanchang, 330006 China; 2 School of Public Health, Jiangxi Provincial Key Laboratory of Disease Prevention and Public Health, Jiangxi Medical College, Nanchang University, Nanchang, 330006 China; 3 National Key Laboratory of Intelligent Tracking and Forecasting for Infectious Diseases, Chinese Center for Disease Control and Prevention, National Institute for Communicable Disease Control and Prevention, Beijing, 102206 China; 4 School of Public Health, Zhejiang Chinese Medical University, Hangzhou, 310053 China; 5 Office of Public Health Studies, University of Hawaii at Manoa, Honolulu, HI 96822 USA

**Keywords:** DLNM, Scrub typhus, Meteorological factors, Geodetector

## Abstract

**Background:**

Scrub typhus is transmitted through vectors and is susceptible to meteorological factors, posing a significant threat to human life and health. Therefore, in this study, the nonlinear relationships between meteorological factors and scrub typhus (ST) and the lag effects of meteorological factors on ST were analyzed, and the explanatory power of these factors on the spatially stratified heterogeneity of ST was evaluated.

**Methods:**

Monthly data on ST cases and meteorological factors were collected in Jiangxi from 2014 to 2023. A distributed lag nonlinear model (DLNM) was used to analyze the lag effects and nonlinear relationships between meteorological factors and ST. Geodetector was conducted using 2023 spatial data to evaluate the explanatory power of meteorological factors and their interactions on the spatially stratified heterogeneity of ST.

**Results:**

A total of 9129 cases of newly diagnosed ST were recorded. The DLNM demonstrated nonlinear relationships between meteorological factors and ST and lag effects of meteorological factors on ST. The influence of temperature, relative humidity, and wind speed on the ST initially increased, peaking at 25.50 °C, 84.80%, and 2.00 m/s, respectively, before decreasing. Precipitation was associated with an increasing risk of ST, whereas pressure tended to decrease risk. Compared with median meteorological values, extreme conditions (such as extremely low temperature, extremely low relative humidity, extremely high pressure, and extremely high wind speed) had a protective effect on the incidence of ST. Conversely, extremely high precipitation and extremely low pressure were associated with an elevated risk of ST. Geodetector analysis revealed the following explanatory power for the spatially stratified heterogeneity of ST: temperature (0.357) > relative humidity (0.351) > pressure (0.275) > precipitation (0.225) > wind speed (0.223). Temperature and relative humidity emerged as the most critical indicators affecting ST. Furthermore, the incidence of ST was driven by the combined effects of multiple meteorological factors.

**Conclusions:**

The incidence of ST in Jiangxi Province is significantly influenced by meteorological factors, with both lag effects and nonlinear relationships. Temperature and relative humidity are the key indicators affecting ST. The consideration of meteorological factors is essential for the prevention and control of ST.

**Supplementary Information:**

The online version contains supplementary material available at 10.1186/s41182-025-00835-0.

## Introduction

Scrub typhus (ST), commonly known as tsutsugamushi disease, is a zoonotic infectious disease caused mainly by the bite of chigger mite larvae carrying *Orientia tsutsugamushi*, *Candidatus Orientia chuto* [1]*, and Candidatus Orientia chiloensis* [2]*.* This disease is characterized by symptoms such as crusted lesions, fever, rash, and enlargement of the lymph nodes at the bite site [3]. In severe cases, it can result in complications, including multiorgan failure involving the heart, liver, spleen, and kidneys, which can lead to death. Globally, it is estimated that 55% of the population lives in regions, where ST is endemic, with approximately one million cases reported annually [4] and a fatality rate of up to 6% among untreated cases [5].

Since the beginning of the twenty-first century, the rapid development of the Chinese economy, accelerated urbanization, increased population mobility, and a warming climate, among other factors, have contributed to the resurgence of ST. An epidemiological study of ST in China from 2006 to 2021 revealed a significant increase in the average annual incidence from 0.088 cases per 100,000 population in 2006 to 1.99 cases per 100,000 population in 2021[6].

As a major vector-borne disease, ST is influenced by climate change, which can affect the survival and reproduction of chiggers and rodents, thereby potentially altering its prevalence [7–9]. Although several studies have investigated the relationships between meteorological factors and the incidence of ST, the exact nature of this connection remains unclear. This ambiguity stems from the use of diverse research methods and varying levels of data analysis, leading to inconsistent conclusions [10].

In recent years, China has experienced a warming trend, marked by a significant increase in the frequency and intensity of extreme weather events, such as extreme temperatures and heavy precipitation. This underscores the importance of studying the impact of extreme weather conditions on ST. Furthermore, there is a notable lack of research on the key meteorological factors driving ST and the extent to which interactions between these factors explain spatial heterogeneity.

This study aims to examine the association between meteorological factors and ST in Jiangxi Province from 2014 to 2023, explore the impact of extreme climatic factors on ST, and analyze the contribution of meteorological factors and their interactions to the spatial heterogeneity of ST. The findings are expected to support the prevention and control of ST, ultimately helping to reduce its future burden.

## Materials and methods

### Study location

Jiangxi Province is located in the southeastern region of China, with coordinates spanning 24°29′14″N to 30°04′44″N and 113°34′36″E to 118°28′58″E. Jiangxi Province encompasses an area of approximately 167 million square kilometers, comprising 100 districts, and has a permanent population of 45.3 million at the end of 2022. Jiangxi Province is characterized by a subtropical monsoon climate, with four distinct seasons. The winter months are mild and humid, whereas the summer season is characterized by high temperatures and precipitation [11].

### Data summary

The data on ST cases that were reported on a monthly basis in Jiangxi Province between 2014 and 2023 were obtained from the China Infectious Disease Surveillance System. The system records comprehensive data on ST patients, including demographic information, such as gender and age, the county where the infection was initially identified, the date of symptom onset, and the date of diagnosis. In this study, the date of symptom onset was used as the primary indicator. The concurrent meteorological data were sourced from the China National Meteorological Information Center and included average temperature, average relative humidity, cumulative precipitation, average pressure, and average wind speed. In this study, a distribution lag nonlinear model (DLNM) was constructed using provincial monthly meteorological factors and ST data from 2014 to 2023, and the geodetector was constructed using spatial meteorological factors and ST data from 100 districts and counties in 2023.

### Assessment of multicollinearity

Spearman's correlation coefficient and variance inflation factor were used to determine the multicollinearity among the meteorological factors, and this study revealed that temperature and pressure exhibited high levels of multicollinearity (Table S1). Consequently, both variables were not included in the same model during the subsequent analysis.

### DLNM

In this study, a distributed lag nonlinear model based on a generalized additive model (GAM) was used to explore the lagged and nonlinear effects of meteorological factors on ST. The model utilized a quasi-Poisson approach to mitigate issues related to excessive data dispersion [12]. The exposure dimension is fitted using a quadratic polynomial without the need to set nodes. The lag dimension adopts a B-spline with 3 degrees of freedom. We considered the life cycles of the mites and rodents and referred to the relevant literature, setting the maximum lag period to 6 months[13]. Cross-basis functions were constructed for each meteorological factor to analyze the lag–exposure–response relationship. When the effects of a specific meteorological factor were examined, all the other meteorological factors were treated as covariates. The median value of each meteorological factor served as the reference point to assess its impact on ST onset. The specific formula is as follows:

1 $$\log [E(Y_{t} )] = \beta + cb(K_{{{\text{t}},6}} ,\beta_{1} ) + s_{1} (x) + s_{2} ({\text{time}})$$

where *Y*_*t*_ is the number of new ST cases in month *t*; *β* denotes the intercept of the whole model; cb() denotes the cross-basis function; and *K* and *X* represent meteorological factors, where *K* is the meteorological factor to be analyzed and *X* is other meteorological factors used to control for confounding; *β*_1_ denotes the estimated value of the effect of *K* in a specific lagged month *t*; 6 denotes the maximum number of lagged months; time is used to adjust for the long-term trend and seasonal characteristics; and both *S*_1_() and *S*_2_() are natural cubic splines, in which the degrees of freedom for *S*_1_() are restricted to between 2 and 6 to avoid overfitting. The degrees of freedom for each variable are then determined on the basis of the principle that smaller the generalized cross-validation (GCV) values are better, with specific values listed in Table S2. The degrees of freedom for *S*_2_() are uniformly set to 3.

Different quantiles of meteorological factors (1st, 5th, 10th, 90th, 95th, and 99th) were defined as extreme meteorological factors. On the basis of the above model, the median of each meteorological factor was used as the reference value to explore the effects of extreme meteorological factors on ST.

### Geodetector

The geodetector is a widely used tool among scholars for impact factor analysis, as it requires fewer assumptions than do traditional statistics [14]. The principle of this method is to divide the independent variables into different strata or categories according to a unified standard and then determine whether there are significant differences in the distribution of Y across these strata or categories[15]. In this study, we employed the quantile method to divide the meteorological factor data into nine equal groups.

Geodetector includes factor detection, which identifies potential influences on a geographic object and calculates the degree of influence of these factors. In addition, it involves interaction detection, which can explain the effect of influencing factors on geographic objects when they interact [16].

### Factor detection

Factor detection is a method that can be used to identify the spatial heterogeneity of ST in Jiangxi Province. It is used to investigate the extent to which meteorological factors contribute to the spatially stratified heterogeneity of ST [16]. The factor detection calculation formula is as follows:

2 $$q = 1 - \frac{1}{{N\sigma^{2} }}\sum\limits_{m = 1}^{L} {N_{m} } \sigma_{m}^{2}$$

where q is the explanatory power of the meteorological factor on ST, and its value range is [0, 1]; the larger the value of *q* is, the stronger the explanation of the factor on the spatial stratified heterogeneity of ST; $N$ and $N_{{\text{m}}}$ are the numbers of districts in the total study area and in the *m*th stratum (*m* = 1,2…,L), respectively; $\sigma^{2}$ and $\sigma_{m}^{2}$ represent the variances in ST in all the districts in the *m*th stratum[15].

### Interaction detector

The interaction detector is capable of detecting the interaction effect of meteorological factors on ST spatially stratified heterogeneity and determining whether the two factors are mutually exclusive or interact with each other. The interaction detector compares and analyses the explanatory power of the two meteorological factors acting individually on the ST spatially stratified heterogeneity *q*(*x*) and *q*(*y*) with the explanatory power of the ST spatially stratified heterogeneity when the two factors are superimposed together *q*(*x*, *y*). It then determines the type of two-factor interaction in accordance with the table of interactions, as shown in Table S3 [17].

### Statistical analysis

In this study, meteorological factors and ST data were collected from 2014 to 2023. Spearman correlation analysis and VIF were used to explore the multicollinearity among meteorological factors, and distributional lag nonlinear models were used to explore the lag effects and nonlinear relationships of the effects of meteorological factors on ST. Based on the spatial data of 2023, the geodetector was used to explore the explanatory power of meteorological factors and their interactions on the spatially stratified heterogeneity of ST. The DLNM was constructed using the dlnm package and mgcv package in R (version 4.3.1), the geodetector was built using the geodetector package in R (version 4.3.1), and some of the mapping was performed using GraphPad 9.0 software.

## Results

**Fig. 1:**
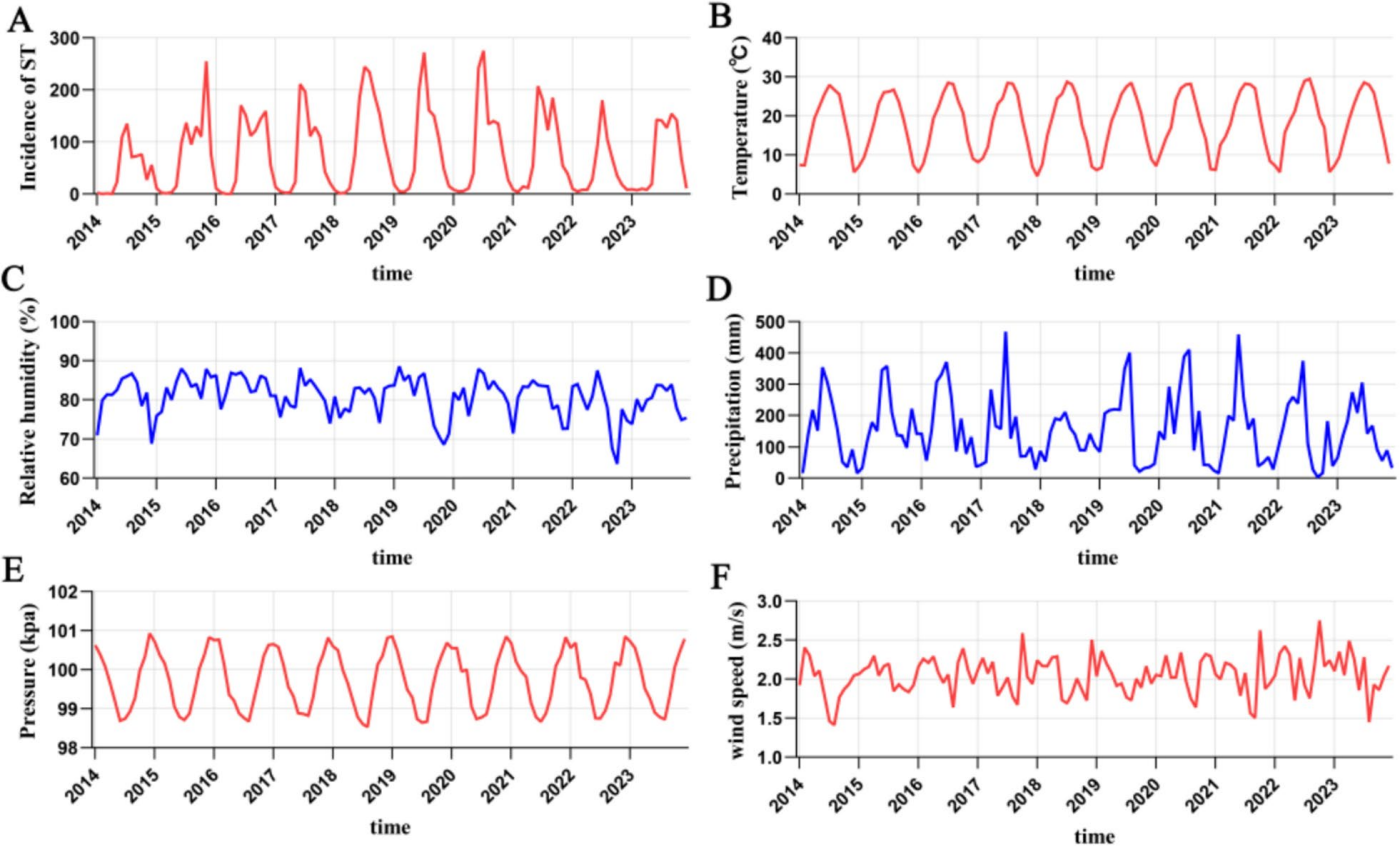
Time series plots of monthly average meteorological factors and scrub typhus in Jiangxi Province from January 2014 to December 2023. **A** Scrub typhus (ST); **B** temperature; **C** relative humidity; **D** precipitation; **E** pressure; **F** wind speed

A total of 9129 ST cases were documented in Jiangxi, China, from 2014 to 2023. A time series plot of meteorological factors and ST is shown in Fig. 1, from which the incidence of ST in Jiangxi Province is shown to be distinctly seasonal. The monthly average number of cases of ST in Jiangxi Province from 2014 to 2023 is shown in Figure S1. The period from June to October is the peak season for ST. Table 1 lists the characteristics of the meteorological factors and ST. The median (interquartile range) temperature, relative humidity, precipitation, pressure, wind speed, and ST cases were 19.178 °C (12.476–25.576), 81.660% (77.970–83.940), 131.358 mm (41.587–210.099), 99.810 kPa (99.030–100.380), 2.049 m/s (1.885–2.212), and 54.000 (9.750–134.250), respectively.

**Table 1:** Description of meteorological factors and ST in Jiangxi Province for 2014–2023

Variables	Mean	SD	Min	P25	Median	P75	Max
Count	76.080	74.878	0.000	9.750	54.000	134.250	275.000
Temperature (°C)	18.215	7.746	4.625	12.476	19.178	25.576	29.468
Relative Humidity (%)	80.830	4.923	63.660	77.970	81.660	83.940	88.570
Precipitation (mm)	141.442	114.802	1.092	41.587	131.358	210.099	467.899
Pressure (kpa)	99.750	0.741	98.530	99.030	99.810	100.380	100.930
Wind Speed (m/s)	2.047	0.248	1.411	1.885	2.049	2.212	2.753

*SD* standard deviation, *Min* minimum, *Max* maximum, *P25* 25th percentile, *P75* 75th percentile

**Fig. 2:**
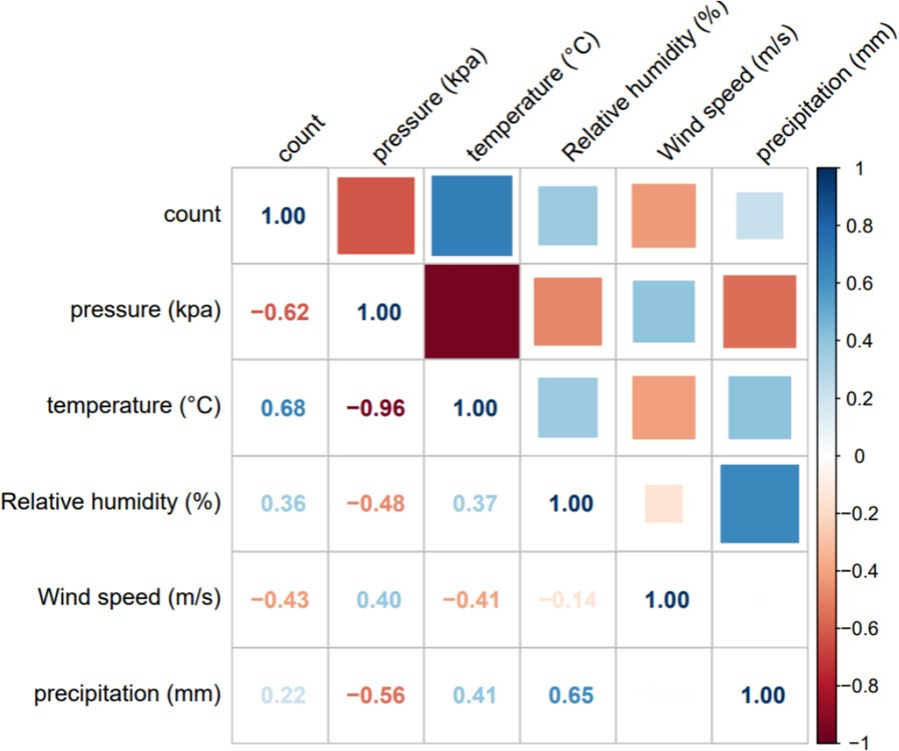
Spearman correlation analysis between meteorological factors and ST in Jiangxi Province for 2014–2023

The Spearman correlation coefficients between the meteorological factors and ST are shown in Fig. 2. Initially, a correlation was identified between all five meteorological factors and ST. In addition, the correlation coefficient between temperature and pressure was -0.96, indicating a strong correlation. To further determine the multicollinearity between meteorological factors, the VIF was calculated (Table S1). Multicollinearity existed between temperature and pressure; thus, both temperature and pressure were not included in the model in the subsequent analysis.

**Fig. 3:**
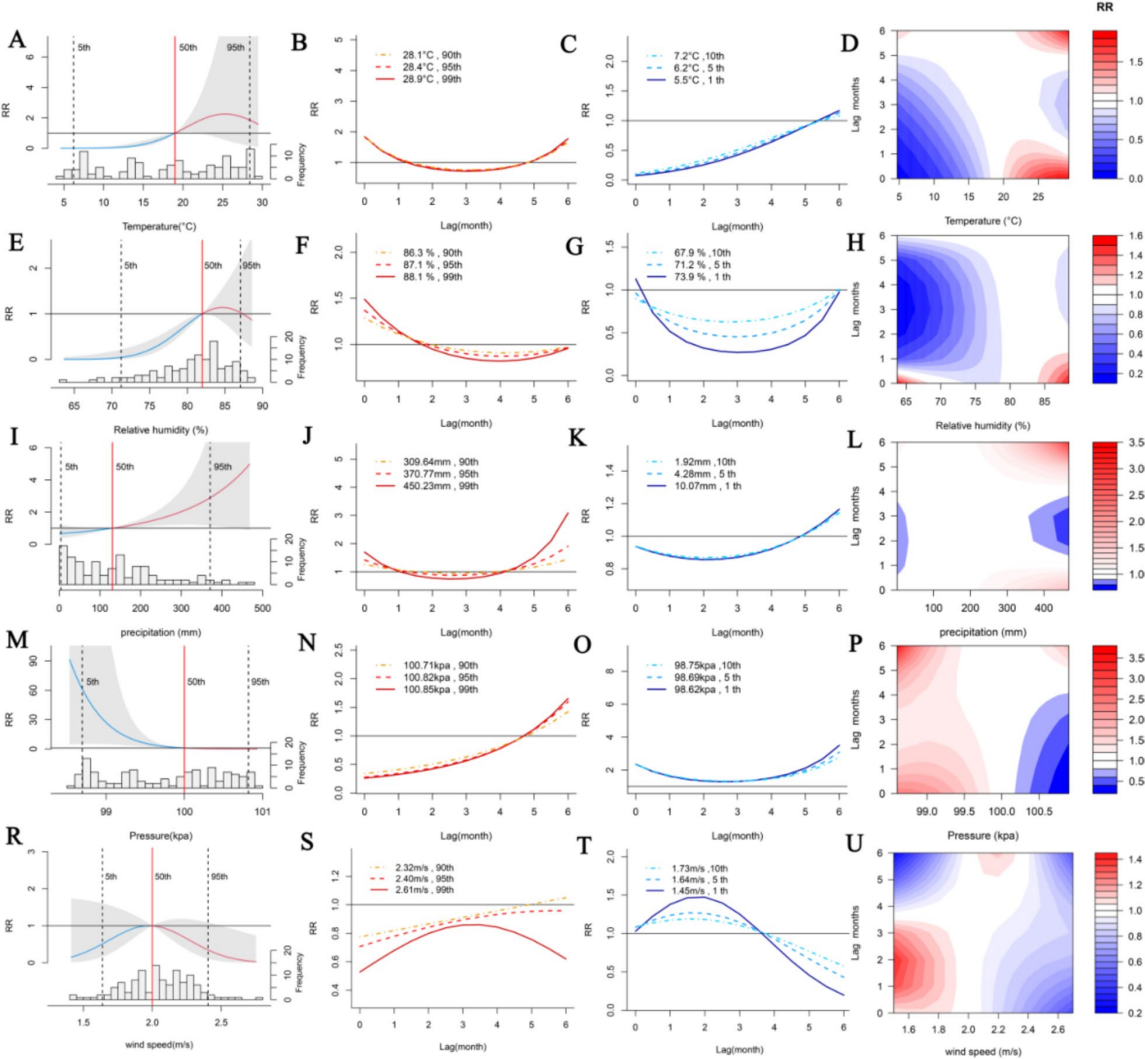
Cumulative and lag effects of meteorological factors on ST in Jiangxi Province for 2014 to 2023. **A**, **E**, **I**, **M**, and **R** represent the cumulative effect plots of temperature, relative humidity, precipitation, pressure, and wind speed, respectively, on scrub typhus (ST). **B**, **D**, **J**, **N**, and **S** represent the effects of extremely high temperature, relative humidity, precipitation, pressure, and wind speed on ST, respectively; **C**, **G**, **K**, **O**, and **T** represent the effects of extremely low temperature, relative humidity, precipitation, pressure, and wind speed on ST, respectively; **D**, **H**, **L**, **P**, and **U** represent the 2D plots of the influence of temperature, relative humidity, precipitation, pressure, and wind speed on ST, respectively; and P90, P95, and P99 are defined as extremely high meteorological factors, whereas P10, P5, and P1 are defined as extremely low meteorological factors

The cumulative and lag effects of the meteorological factors on the impact of ST are shown in Fig. 3. The nonlinear relationship and lag effect of the meteorological factors on the ST are shown in Fig. 3A, E, I, M, and R, respectively, and the cumulative effects of the meteorological factors on the risk of ST with lags of 0–6 months are shown in Fig. 3. Among them, the impacts of temperature, relative humidity, and wind speed on the ST initially increase but then decrease, reaching their maximum values at 25.50 °C, 84.80%, and 2.00 m/s, respectively. The risk of ST incidence from precipitation has been increasing, whereas the risk of ST incidence from pressure has been decreasing.

As illustrated in Fig. 3B, C, F, G, J, K, N, O, S, and T and Table 2, the impact of extreme meteorological conditions on ST is clearly evident. The effects of extreme meteorological factors on ST are essentially similar across different quantiles. In addition, a lag effect was observed in different lag months, with varying degrees of impact on ST. As illustrated in the table, in comparison with the median temperature, the effects of extremely low temperatures on ST were protective, with the lowest RR value observed at the 1st percentile (temperature value = 5.539 °C; RR = 0.001; 95% CI 0.000–0.013). The data indicate that compared with the median relative humidity, the extremely low relative humidity was protective of ST. The lowest RR value of relative humidity was observed at the 1st percentile (relative humidity value = 67.876%, RR = 0.007; 95% CI 0.001–0.117). The occurrence of extremely high precipitation was found to increase the risk of ST development in comparison with that associated with median precipitation. The highest RR value of extremely high precipitation was observed at the 95th percentile (precipitation value = 370.774 mm; RR = 2.887; 95% CI 1.166–7.143). Compared with median pressure, extremely low pressure was associated with an increased risk of ST. The lowest RR value of pressure was observed at the 1st percentile (pressure value = 98.616 kPa; RR = 71.930; 95% CI 34.553–149.7). In contrast, extremely high pressure had a protective effect on ST, with the highest RR value of pressure at the 99th percentile (pressure value = 100.851 kPa; RR = 0.029; 95% CI 0.015–0.053). The data indicate that extremely high wind speed was protective of the ST at the 99th percentile in comparison with the median wind speed (wind speed value = 2.618 m/s; RR = 0.090; 95% CI 0.009–0.870).

**Table 2:** Cumulative impact of extreme meteorological factors on ST in Jiangxi Province for 2014–2023

		P1	P5	P10	P90	P95	P99
Temperature (°C)	value	5.539	6.212	7.182	28.08	28.409	28.91
	RR (95% CI)	**0.001** **(0.000, 0.013)**	**0.001** **(0.000, 0.017)**	**0.001** **(0.000, 0.028)**	1.926 (0.194,19.095)	1.858 (0.169,20.473)	1.735 (0.131,22.919)
Relative Humidity (%)	value	67.876	71.236	73.912	86.335	87.045	88.095
	RR (95% CI)	**0.007** **(0.001, 0.117)**	**0.043** **(0.008, 0.214)**	**0.139** **(0.056, 0.345)**	1.083 (0.594,1.975)	1.030 (0.487,2.179)	0.918 (0.333,2.531)
precipitation (mm)	value	1.918	4.276	10.066	309.648	370.774	450.231
	RR (95% CI)	0.667 (0.366, 1.216)	0.671 (0.374, 1.205)	0.682 (0.394, 1.179)	**2.120** **(1.220, 3.684)**	**2.887** **(1.166, 7.143)**	4.478 (0.941, 21.320)
Pressure (kpa)	value	98.616	98.695	98.748	100.713	100.82	100.851
	RR (95% CI)	**71.93** **(34.553, 149.738)**	**56.476** **(29.535, 107.994)**	**49.197** **(26.892, 90.001)**	**0.067** **(0.043, 0.102)**	**0.044** **(0.026, 0.074)**	**0.029** **(0.015, 0.053)**
Wind Speed (m/s)	value	1.450	1.638	1.732	2.322	2.405	**2.618**
	RR (95% CI)	0.197 (0.023, 1.718)	0.515 (0.166, 1.600)	0.701 (0.327, 1.502)	0.506 (0.223, 1.149)	0.354 (0.115, 1.089)	**0.090** **(0.009, 0.870)**

The P90, P95, and P99 of the meteorological factors are defined as extremely high, whereas the P10, P5, and P1 are defined as extremely low

*ST* scrub typhus, *RR* relative risk, *P1* 1st percentile, *P5* 2nd percentile, *P10* 10th percentile, *P90* 90th percentile, *P95* 95th percentile, *P99* 99th percentile

**Fig. 4:**
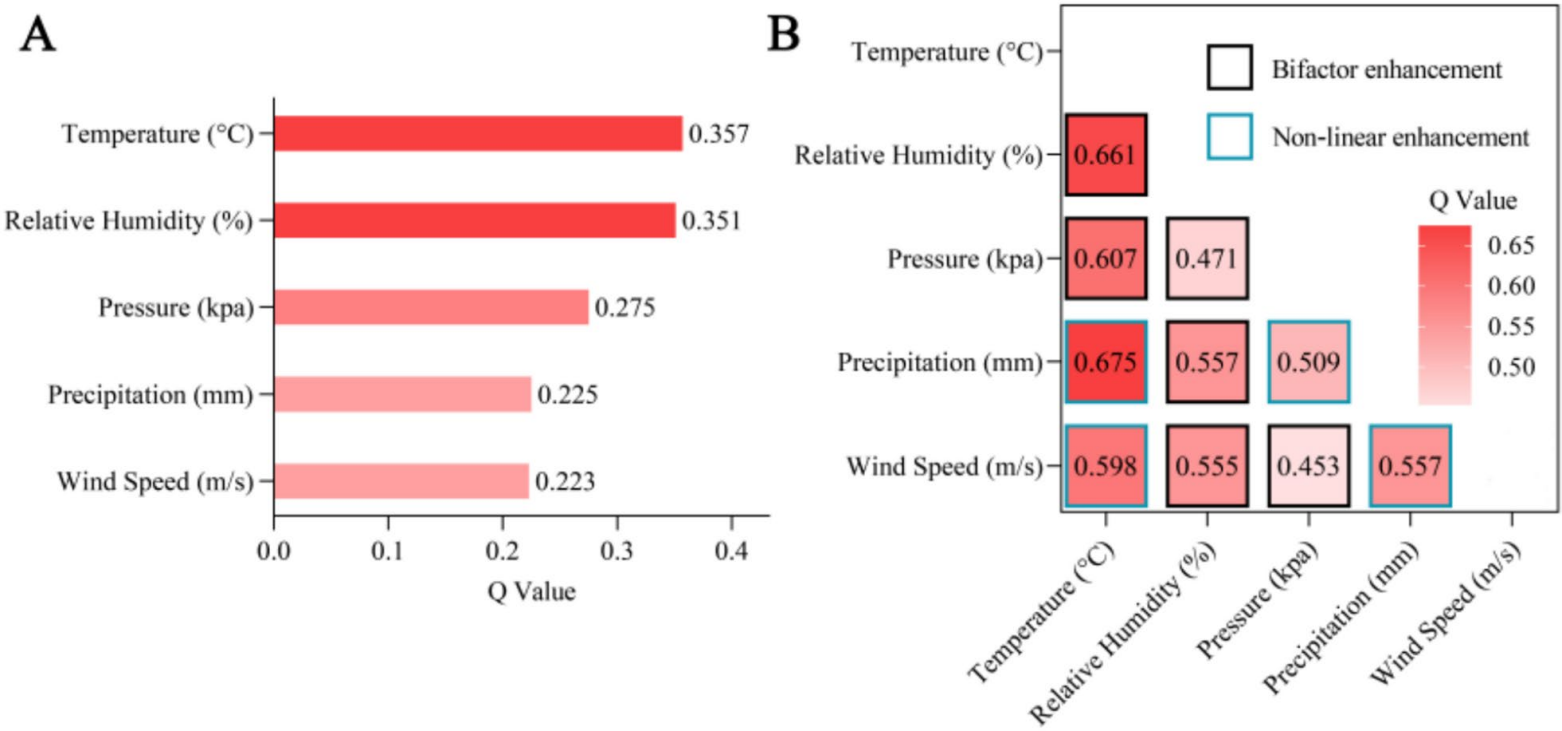
Explanatory power of the geodetector for meteorological factors and their interactions on ST in 2023. **A** Factor detection; **B** interaction detection

Factor detection was used to calculate the strength of influence of the meteorological factors on ST (Fig. 4A). All five meteorological factors passed the test of significance at the 0.05 level. The explanatory power of the five meteorological factors on the spatially stratified heterogeneity of ST, in descending order, was temperature (0.357), relative humidity (0.351), pressure (0.275), precipitation (0.225), and wind speed (0.223), with temperature and relative humidity having relatively high explanatory power on the spatially stratified heterogeneity of ST. Interaction detection was used to calculate the interaction effect of meteorological factors on ST (Fig. 4B). We found that the types of interaction between meteorological factors were all enhanced, with a bifactor enhancement dominating. The explanatory power of the interaction between any two meteorological factors is greater than that of the original single factor, and the ST in the Jiangxi Province area is caused by the combined effect of several meteorological factors.

## Discussion

In this study, the associations between meteorological variables were investigated using meteorological and ST data from 2014 to 2023 in Jiangxi Province. Our results revealed that the impact of meteorological factors on ST follows a lagged and nonlinear pattern. Extreme meteorological conditions, compared with the median values, were found to have a significant effect on ST. Notably, this study was the first to apply a geodetector to analyze the explanatory power of meteorological factors and their interactions on the spatially stratified heterogeneity of ST. The findings revealed temperature and relative humidity as the primary influencing factors, with interactions between temperature and other variables significantly enhancing the explanatory power of the spatial heterogeneity of ST in Jiangxi Province.

Our findings indicated that the risk of ST positively correlated with increasing temperature, peaking at 25.50 °C before it gradually decreased. These findings align with those of the study conducted by Lu et al. [18]. Temperature significantly influences the activities of rodents, mites, and humans [19]. Research has shown that the life cycle of the primary mite species in southern China, *L. deliense*, can be completed within a temperature range of 13 ± 1 to 35 ± 1 °C [20], with 23 ± 1 °C being the optimal temperature for its development and reproduction. Such optimal temperatures may accelerate the reproduction and development of rodent hosts and mites, increasing the potential for the development of ST [18, 21]. Moreover, favorable temperatures can promote agricultural and recreational activities, highlighting the risk of human exposure [22]. Conversely, extreme temperatures may reduce the willingness to engage in such activities, thus reducing the risk of contracting ST.

Several studies have reported a positive association between relative humidity and the incidence of ST [23, 24], and our findings are generally consistent with these observations. The biological basis for this phenomenon can be attributed to the following factors: under low-humidity conditions, adult chiggers cease laying eggs, whereas increased relative humidity creates an environment more favorable for mite growth [25, 26]. In addition, prior research has demonstrated a positive association between precipitation and the prevalence of chiggers, which aligns with our results [24, 26]. Our data indicate that the likelihood of ST occurrence increases with increasing precipitation. This can be attributed to enhanced vegetation growth driven by precipitation, ultimately leading to higher rodent densities [27]. Some studies also indicate that when precipitation exceeds a certain threshold, the risk of ST decreases [28, 29]. High precipitation prevents people from farming or engaging in outdoor activities, reducing their exposure to chiggers and thereby decreasing the incidence of ST.

The results indicate that the wind speed initially increases and then decreases, reaching its maximum at 2.00 m/s. These findings are in general agreement with the findings of Lu et al. [18]. Wind speed may influence chigger egg laying, as windy weather is often accompanied by precipitation, which can affect the activities of individuals who are engaged in agricultural work or who are sitting on grass. Currently, research explaining the potential mechanisms by which wind speed affects ST is limited. Future studies should further investigate the mechanisms underlying the impact of wind speed on ST.

The negative impact of pressure on ST is roughly opposite to that of temperature, which is consistent with the findings of a previous study conducted in Guangzhou [26]. One possible explanation is that high pressure is detrimental to mites [18]. High-pressure environments are typically accompanied by lower humidity and temperature, as well as reduced sunlight exposure, which are meteorological conditions unfavorable for the survival of mites; this may lead to a decrease in the number of ST cases[26]. In addition, our findings revealed a strong negative correlation between pressure and temperature. As temperature was not controlled during subsequent model construction, the reliability and validity of the observed effect of pressure on the ST remain uncertain. Further research is needed to clarify these findings.

To the best of our knowledge, this study is the first to apply a geodetector to examine the impact of meteorological factors on ST. The results show that temperature and relative humidity are the key indicators affecting ST, which is consistent with the conclusions of Zheng et al. [30]. This may be attributed to the critical role of temperature and humidity in the development and reproduction of mites [31]. Low temperatures extend the developmental cycle of mites while increasing their mortality prior to the completion of their reproductive cycle. Similarly, humidity is a crucial determinant of mite density, as mites depend on water vapor for survival [32]. Moreover, this study highlights that the prevalence of ST in Jiangxi Province is influenced by a complex interplay of meteorological factors. These factors affect human health in various ways, including influencing the proliferation of pathogens, the growth and reproduction of vectors, and human social behavior [33]. The occurrence of ST is closely linked to meteorological variables, such as temperature, humidity, and precipitation, which collectively support the growth, reproduction, and activity of the ST vector, thereby increasing the risk of ST.

Several limitations should be acknowledged in this study. First, the data were derived from a passive reporting surveillance system, which is prone to under-reporting and misreporting because of regional variations in ST detection, diagnosis, and treatment capabilities. Second, while the study focused on meteorological factors, other influences, such as socioeconomic factors, ST vector densities, host densities, and human behavioral patterns, were not considered. Finally, this study analyzed the total number of ST cases in Jiangxi Province, and the findings may not be directly generalizable to individuals or other populations because of group-specific differences. To avoid ecological fallacies, further experimental studies are needed to validate these findings.

## Conclusion

This study revealed a lagged and nonlinear relationship between meteorological factors and ST, highlighted the impact of extreme meteorological factors on ST, examined the extent to which these factors and their interactions influence ST, and revealed key meteorological contributors. This study offers a theoretical basis for designing effective ST prevention and control strategies. Future studies will delve deeper into the influence of socioeconomic and other environmental factors on ST.

## Supplementary Information

Additional file 1.

## Data Availability

Data will be available from the corresponding author on reasonable request.
