# Supplementary material for: Influence of meteorological factors on scrub typhus in Southeast China: a study across 100 districts in Jiangxi Province
Source: Trop Med Health. 2025 Nov 7;53:157. doi: 10.1186/s41182-025-00835-0 (PMC12595867; doi:10.1186/s41182-025-00835-0)
Supplement: Supplementary file 1 — Additional file 1. [file 41182_2025_835_MOESM1_ESM.docx]

Table S1 Variance inflation factor among meteorological factors

|  | meteorological factors | | | | |
| --- | --- | --- | --- | --- | --- |
|  | Temperature (℃) | Pressure  (kpa) | Relative Humidity (%) | Precipitation (mm) | Wind Speed (m/s) |
| VIF | 17.348 | 21.815 | 1.847 | 2.611 | 1.303 |

Table S2 The GCV values corresponding to different degrees of freedom of the spline function in the Cross basis

| Cross basis | Other meteorological factors | Degree of freedom | | | | |
| --- | --- | --- | --- | --- | --- | --- |
|  |  | 2 | 3 | 4 | 5 | 6 |
| Temperature (℃) | Wind Speed | 12.093 | **11.939** | 12.119 | 11.538 | 11.579 |
|  | precipitation | 11.939 | 11.913 | **11.81** | 11.834 | 12.179 |
|  | Relative Humidity | 11.81 | **11.384** | 11.576 | 11.473 | 11.702 |
| Relative Humidity (%) | Wind Speed | 34.112 | **31.423** | 32.119 | 31.965 | 32.445 |
|  | Temperature | 31.423 | 31.539 | 31.514 | **31.154** | 31.894 |
|  | precipitation | **31.154** | 31.803 | 31.903 | 32.497 | 33.24 |
| Wind Speed (m/s) | Temperature | **28.923** | 28.983 | 29.625 | 30.129 | 30.564 |
|  | Relative Humidity | **28.923** | 29.360 | 29.861 | 30.238 | 30.859 |
|  | precipitation | **28.923** | 29.455 | 30.087 | 30.190 | 30.886 |
| Pressure(kpa) | Wind Speed | 8.546 | 8.215 | 8.230 | **7.732** | 7.584 |
|  | precipitation | 7.732 | 7.485 | 7.253 | **7.219** | 7.232 |
|  | Relative Humidity | 7.219 | 6.342 | 6.295 | 6.050 | **6.000** |
| precipitation (mm) | Temperature | **29.750** | 30.239 | 30.878 | 30.716 | 31.032 |
|  | Relative Humidity | **29.750** | 30.354 | 30.658 | 30.126 | 30.670 |
|  | Wind Speed | 29.750 | **28.462** | 29.060 | 29.296 | 29.675 |

Note: Bold represents the GCV value corresponding to the final set degree of freedom

Table S3 Interaction between Explanatory Variables

| Expression | Interaction |
| --- | --- |
| q(x, y)<Min(q(x), q(y)) | non-linear Weaken |
| Min(q(x), q(y))<q(x, y)<Max(q(x), q(y)) | unitary-non-linear Weaken |
| q(x, y)>Max(q(x), q(y)) | bifactor enhancement |
| q(x, y)=q(x)+q(y) | Independent |
| q(x, y)>q(x)+q(y) | non-linear enhancement |


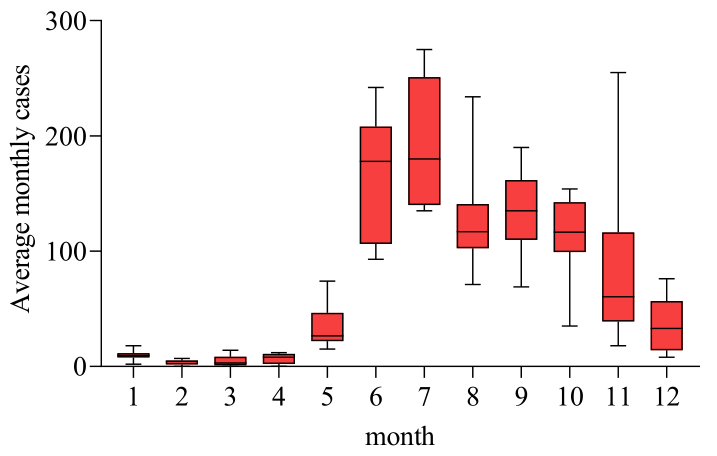


Figure S1 The monthly incidence of ST in Jiangxi Province from 2014 to 2023
